# Leveraging technology for health services continuity in times of COVID-19 pandemic: Patient follow-up, and mitigation of worse patient outcomes

**DOI:** 10.7189/jogh.11.05024

**Published:** 2021-12-25

**Authors:** Elizabeth Muli, Rebeccah Waithanji, Moses Kamita, Tabither Gitau, Ishmael Obonyo, Sharon Mweni, Faith Mutisya, Peter Kirira, Ancent Nzioka, Jonine D Figueroa, Francis Makokha

**Affiliations:** 1 Department of Computer Science and Technology, Technical University of Kenya, Nairobi, Kenya; 2 Directorate of Research and Innovation, Mount Kenya University, Thika, Kenya; 3 Cancer Care and Research Centre, Machakos County, Machakos, Kenya; 4 Department of Health Machakos County, Machakos County, Kenya; 5 The University of Edinburgh, Usher Institute, Centre for Global Health, Edinburgh, UK

## Abstract

**Background:**

Since the outbreak of the COVID-19 pandemic in Wuhan, China, which has now spread globally, the health systems continue to face challenges in the provision of health care, there is a risk of exposure for both the physicians and the patients. While there is significant progress in the adoption of technology in health care. This study sought to examine the adverse effects of the measures put in place by the government to curb the spread of COVID-19 and come up with an intervention to prevent worse outcomes for chronic conditions.

**Methods:**

Booking registers for four specialty clinics in Machakos Level 5 Hospital were reviewed to identify patients who missed clinic appointments for follow-up. An automated data collection tool (ODK-collect) was used for data collection. COVID-19 Machakos App was developed to facilitate follow-up and referral of patients to the nearest facilities, capturing and posting of information in real-time to a central database. The mobile App also facilitated the tracking of patients and aided doctors to give feedback on whether the patients reported to the referred facilities. The doctors were also able to capture doctors’ notes on the patients' status while ensuring the confidentiality and privacy of the patients. An interactive dashboard was developed to generate analytics reports and summaries to monitor clinic attendance and trends in the provision of health care during the pandemic period.

**Results:**

Register data showed 977 (81.5%) out of a total of 1199 patients had missed their scheduled appointments. Among the 977, 746 (76%) were residents of Machakos County and qualified for follow-up. Missed appointments varied by clinic: Cancer Clinic 12 (1.6) %), Diabetes Clinic 212 (28.4%), Hypertension 293 (39.3%), and Paediatrics Clinic 229 (30.7%). Contact was made and follow-up was attempted for 746 patients, of which 453 patients (60.7%) were successful. The follow-up distribution of the 453 patients varied by the clinic as follows: Cancer Clinic 10, Diabetes Clinic 146, Hypertension 185, and Paediatrics Clinic 112. During the follow-up process, 331 patients from diabetes and hypertension clinics were requested to choose a preferred or nearby facility to be referred to. 191 (58%) patients chose Machakos Level 5 Hospital as their preferred facility and 137 (41%) patients chose to be referred to level 3 or 4 hospitals within the County. Three deaths were reported from the medical (Hypertension) clinic. Through the developed App, a total, 82 (60%) patients out of the 137 were reviewed at the referral facilities jointly with a specialist at Machakos Level 5 Hospital. For the duration of the study, some patients reported worse conditions by the time of review after missing scheduled appointments.

**Conclusions:**

This intervention demonstrated that mobile phone technology could be leveraged to provide specialty treatment services remotely to mitigate against worse patient outcomes. The study reveals that there is a dire need to integrate technological interventions in the provision of health care services to ensure continuity.

COVID-19 was declared a Public Health Emergency of International Concern on 30 January 2020 and a pandemic on 11 March 2020 [1]. Center for Disease Control (CDC) reported human transmission to be through infected air droplets resulting from coughing or sneezing, contact with infected surfaces, and in turn touching eyes, nose, and mouth [2]. The COVID-19 pandemic has had an unprecedented impact on deaths but also on public health care services. To curb the spread of COVID-19 most country's authorities responded by implementing measures such as; lockdowns, local and international travel restrictions, work hazard controls, and closure of facilities such as churches, schools, restaurants, and parks among others [3]. As of 14 August 2020, 21 128 670 cases of COVID-19 had been reported and 758 391 deaths [4]. Kenya reported its first case of COVID-19 on 13 March 2020 [5]. Immediately, the Kenyan government put in place measures to curb the spread of the virus in the country. These measures included campaigns to maintain high standards of hygiene (handwashing and use of sanitizers), social distancing, the closing of educational institutions, closure of bars and hotels, ban on religious gatherings, open-air markets fumigation, and limit on the attendees of burial and wedding ceremonies. National wide 7 pm to 5 am curfew was reinforced, travel restriction to and from high risks counties (Nairobi, Mombasa, Kilifi, and Kwale), reduced the capacity of all public transport to half the capacity and enforcing the compulsory wearing of masks in public spaces [6]. Despite these measures, by end of August 2020, Kenya had registered the highest number of COVID-19 cases in East Africa [7]. This could be attributed to the fact that in major cities like Nairobi, two-thirds of the 4.4 million people are crowded in informal settlements that lack basic services, and a whole family can live in a single room [8]. These precautionary measures helped in containing secondary spread within communities but resulted in negative socio-economic impacts on the Kenyan people at large; job losses, closure, and losses in businesses slashed working hours and salaries resulting in low outputs [9].

In health care provision, these measures have presented unprecedented challenges for both health care workers and patients. According to a report by Daily Nation, changes in the transport sector [10] resulted in increased costs of transport. This left patients without many options, as they could not afford the cost of transport. As a developing country loss of jobs and unpaid leaves increased the burden of seeking health care because some of these people were the breadwinners in the families they represent. Additionally, imposed curfews and transport restrictions limited the ability of patients to travel to referral health facilities located far away from patients’ residences [10] thus the need for telemedicine. Worse outcomes of chronically ill patients have been reported as a result of missed appointments and lack of medical care [11].

Considering all of the above, there is a dire need to explore innovative ways to deliver health care services as we adhere to the government measures and set guidelines to curb the spread of COVID-19. Telemedicine is not all new technology and has evolved to become part of health care services delivery [12]. The use of telemedicine can reduce the exposure of staff to ill persons, minimize the use of personal protective equipment (PPE), and decongest the health facilities. The COVID-19 pandemic has accelerated the use of telemedicine and boosted its use by both medics and patients [13]. It has enhanced health services continuity, bringing both the patient and the medic closer without the need to travel [14]. This has also enhanced maintenance of social distancing thus reducing risks of exposure to the disease for both the physician and the patient. A study conducted by CDC shows that telemedicine has been achieved by a real-time audio-video call where patient data are collected and stored in the patient’s portal and used as a point of reference in the future [15].

The major focus of this study was to leverage mobile technology to acquire and use data on patient attendance on hospital admissions to describe the impact of the COVID-19 pandemic on access to health care clinics and hospital services to improve patient access to health care services during the COVID-19 pandemic. In Kenya, the use of telemedicine has not been adopted widely and there is a lack of scientific evidence on the benefits of e-health interventions, how they work under different conditions within health systems remains a major limitation to evidence-based policy and programming [16]. In Kenya, the use of telemedicine has not been adopted widely and there is a lack of scientific evidence on the benefits of e-health interventions, how they work under different conditions within health systems remains a major limitation to evidence-based policy and programming [16]. It was hypothesized that leveraging technology reduces the adverse effects of the measures put in place to curb the spread of COVID-19 and mitigate worse patient outcomes.

We adopted a descriptive approach because descriptive epidemiology is an important tool in managing and responding to public health crises. While high-income countries like the United States (US), Europe, and the United Kingdom (UK) have established reporting of hospital admissions, cancer registries, and clinic attendance, we would note that in Low and Middle-Income Countries (LMIC’s) this is not well established. Hence we were able to collect, compare and descriptively quantify hospital admission, clinic attendance, and patients who had missed appointments using mobile technology in a setting where limited access to data exist. Further, our study was aimed at determining if mobile technology could be adopted to enable patients to access health services at more local lower-tier hospitals closer to their proximity in consultation with experts at Machakos level 5 due to restrictions on travel and other barriers to accessing health care. In Kenya, public health facilities are categorised into six levels based on the type of services offered. Level 1 facilities are community health units managed by community health volunteers who monitor disease at the household level. Level 2 are health dispensaries that act as first aid drug dispensing centres at the community level and level 3 facilities offer maternity and non-specialised treatment services. Level 4 facilities offer surgery and some special treatment services while level 5 facilities can offer all specialty services including intensive care units (ICU) and serve as county referral facilities. Level six hospitals are national referral facilities meant to offer services that are not offered at level five (5) hospitals.

## METHODS

### Study site

**Figure 1:**
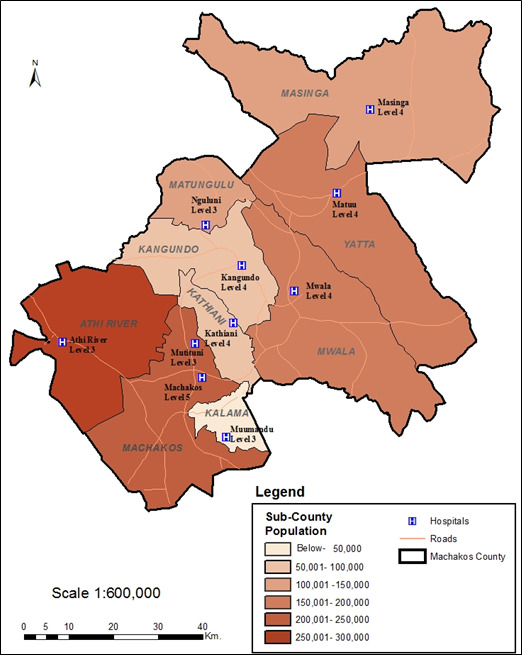
Map of Machakos County showing the nine (9) Sub-counties, the selected health facilities, the catchment population, and bed capacity for each health facility.

The study was conducted in all the nine (9) sub-counties of Machakos County, namely, Kangundo, Machakos, Athi-River, Yatta, Kathiani, Mwala, Matungulu, Masinga, and Kalama (**Figure 1**). This study focused on the patients who were booked for specialty clinics (cancer, diabetes, hypertension, and paediatric clinics) in Machakos Level 5 Hospital. One health facility (**Table 1**) either a level 3 or 4 facility was selected for referring the patients who selected the facility as their preferred nearest facility. The recruitment of patients considered all the patients who had scheduled clinics in cancer, diabetes, hypertension, and paediatric clinics from March to May 2020.

**Table 1:** Selected health facilities at each sub-county in Machakos County, their catchment population, and bed capacity

Sub-county	Health facility	Catchment population	Bed capacity
Kangundo	Kangundo	37 000	176
Machakos Town	Mutituni	10 985	4
Athi-River	Athi-River	38 000	11
Yatta	Matuu	36 913	42
Kathiani	Kathiani	16 814	129
Mwala	Mwala	17600	22
Matungulu	Nguluni	26 285	14
Masinga	Masinga	22 885	10
Kalama	Muumandu	7620	13

### Study design

**Figure 2:**
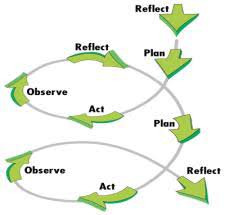
Action research spiral model.

Action Spiral Model was used to guide the design process (**Figure 2**) of developing the Mobile Application and the analytics dashboard. The Action Spiral Model is a developmental process model that consists of the following cycles Reflect, Plan, Act, Observe then reflect again. The cycles represent an incremental improvement approach for rapid cycle change that was used to design, test, and disseminate the interventions. This approach divides the development process into several steps for improvement, evaluation, and reflection.

### Ethical consideration and approvals

This study obtained approval from Mount Kenya University Ethical Review Committee number *683* and a research permit from the National Commission for Science, Technology, and Innovation (NACOSTI) number *NACOSTI/P/20/4830*. The Department of Health of Machakos County Government authorised this study. The research team was provided with patients' files under the supervision of authorised health records officers and this study only collected data without identifiable personal information from the patients’ files. To ensure patients' confidentiality the nurses contacted all the patients during follow-up and linking. All connections and data transmissions to and from the database were done via a secure socket layer (SSL). This ensured that all the data was encrypted using an SSL certificate during transmission to prevent eavesdropping.

### Automation of data collection tools

Open Data Kit (ODK) tool automated the data collection tools and it was used to collect and manage data transmission. All the collected data was transmitted to a secure central MySQL database via a secure link. The database is one of the backend components of the Interactive dashboard and the Mobile Application.

### Application Integration and Features

**Figure 3:**
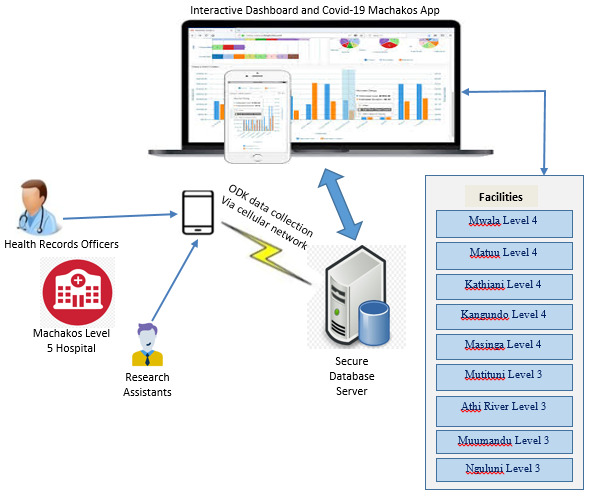
Architectural model of the application.

The Application is comprised of two parts, which are the mobile application (COVID-19 Machakos App), and the interactive dashboard as illustrated in (**Figure 3**).

**Figure 4:**
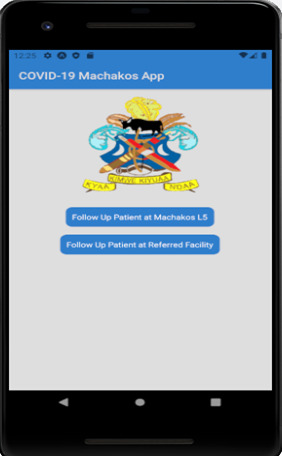
The mobile application used to link patients showing the home screen.
  The two navigation buttons on the App home screen function are:
    - Patient Follow up at Machakos Level 5
  This feature facilitated the capture of patients' details during phone follow-up at Machakos Level 5 hospital. The details included the date of follow-up, reasons for not attending clinic appointments, patient’s preferred facility, referral facility, and the date of reporting to the referral facility. During the data capture in the App, the unique study identifier was used to search for patient details from the database as entered in the ODK Collect then the patients’ record was verified before updating the follow-up information.
    - Follow-up at the referred facility
  This feature facilitated the follow-up of the patient at the referred facility. The process began with the doctor searching for a patient using the unique study identifier. Once a patient's record was found, the study identifier, age, gender, referral facility, and referral date are displayed. The doctor then captured more information on the patient such as whether the patient reported to the referral facility, whether the patient was reviewed and the doctor’s brief notes/feedback on the patient without infringing on the patient's privacy. This information was then updated to the database.
- ***Interactive System Dashboard:*** This system facilitated a real-time generation of report summaries and key metrics to enable end-users to visualize, filter, and get a deeper understanding of the key performance indicators (KPIs) of the research. The dashboard is comprised of algorithms, data analytics, and visualization tools to simplify the complex data sets into meaningful reports and summary graphs. Some of these reports included inpatient and outpatient trends, clinic attendance, and analysis by age, and gender, among others.

- ***Secure database server*** –This server hosted patients’ data on a secure cloud platform and all connections and data transmissions to and from the database were done over SSL. This ensured that data between the server and applications were encrypted using the SSL certificate to prevent eavesdropping.
- ***Mobile application*:** The mobile application (COVID-19 Machakos App) facilitated the linking of patients who missed their clinic appointments to the nearest lower facilities (either Level 3 or Level 4). The COVID-19 Machakos App was implemented using React Native mobile development framework. This App was customized to adapt to our requirement changes easily and meet the specific need of our research to refer patients to lower facilities close to their residence. We were also able to implement tight security measures to make it secure and harder for hackers to infiltrate. Existing applications in the health sector do not have a feature for the downward referral of patients to lower facilities. The linking of patients between the referral facility and specialty clinics at Machakos Level 5 Hospital using the App was done in two steps as shown in the App home screen (**Figure 4**).

### Identification of patients for follow up

Four clinics, cancer, diabetes, medical (hypertension clinic), and paediatrics clinics were purposively selected out of fifteen (15) specialty clinics at Machakos Level 5 Hospital. This was because patients who visit these clinics have chronic conditions that require time-to-time doctor’s review and follow-up. Patient booking registers at the specialty clinics for March, April, and May 2020 were obtained. From the registers, a list with the patients’ date of appointment at the specialty clinic and the patient’s hospital number was generated for use by health records officers (HROs) to retrieve patient files. All patients with a clinic appointment in the three months of study were included. Each patient was assigned a unique study identifier to ensure confidentiality and privacy. Once retrieved, the files were handed over to the study team members who had undergone training on research ethics and data abstraction using a pre-set patient data abstraction form.

The abstraction form was used to extract redacted patient data from patient files. Data captured included the patient’s gender, age, residence, main condition, and co-morbidity(-ies), last date seen at the specialty clinic, and next date of appointment. The abstracted data was then captured into the ODK Collect application for transmission to the database. The data was analysed to determine those patients who required follow-up based on missed clinic appointments and if they were residents of Machakos County. Patients who were not residents of Machakos County were excluded from the follow-up since other counties were not in the scope of our study. Also, patients who had not provided their contacts did not qualify for follow-up regardless of their county of residence.

### Patient follow-up

Patient follow-up was done via phone call by a nursing officer and a health records officer to elucidate the patient’s status, reasons for missing the clinic appointment, and the nearest level 3 or 4 health facility in their sub-counties. This information was captured using the COVID-19 Machakos App. The information was submitted to the database, for patients who were not reachable through their primary mobile phones, their next of kin were contacted.

### Patient referral

After determining the patient’s nearest facilities and reasons for missing scheduled appointments, a list was generated based on the patient’s nearest Level 3 or 4 facilities. The nurses contacted these facilities informing them of the expected number of patients and sought advice on a preferred day to jointly review the referred patients in consultation with a specialist at Machakos Level 5 Hospital. The patients were then contacted and informed of the appointment date at the referral facility. At the referral facilities, patients went through triage to prioritize their care. On getting to the consultation room, the clinical/medical officer reviewed the patient in consultation with the specialist at Machakos Level 5 through a phone call. The Clinical/Medical Officer briefed the specialist on the patients’ status for recording on the patient’s file at Machakos Level 5. In return, the specialist shared the treatment history and plan based on information in the patient records at Level 5. A decision on the management plan of the patient was made jointly after discussing the review then the clinical/medical officer at the referral facility captured the patient data using the COVID-19 Machakos App.

## RESULTS

This study aimed at assessing the adverse effects of the measures put in place by the government to curb the spread of COVID-19 and come up with an intervention to prevent worse outcomes for chronic conditions such as cancer, diabetes, and hypertension. Quick acquisition and determining if mobile technology could assist patients was an important aim. Through this, we were able to obtain data and reach patients in such a short timeline and provide them with assistance. This was a great achievement within such a short time given the crisis. 1199 files for patients scheduled to attend four (4) specialty clinics between March and May 2020 at Machakos Level 5 Hospital were retrieved for data abstraction. These clinics were cancer, diabetes, hypertension, and paediatric clinics. The distribution of the files among the selected clinics was as follows: 76 from the cancer clinic, 315 from the diabetes clinic, 448 from the hypertension clinic, and 360 from paediatrics clinic.1002 (83.6%) patients were residents of Machakos county and 197 (16.4%) were from other neighbouring counties. 977 (81.5%) out of the 1199 patients missed their scheduled appointments. Among these, 746 (76%) patients were residents of Machakos County and qualified for follow-up. The remaining 231 (24%) patients did not qualify for follow-up because 146 (63.2%) were not residents of Machakos County and 85 (36.8%) did not have contact information in their records. The patients' follow-up distribution is as follows: Cancer Clinic 12 (15.7%), Diabetes Clinic 212 (67.3%), Hypertension 293 (65.4%), and Paediatrics Clinic 229 (63.6%). During the follow-up process, patients were contacted by a health records officer or a nursing officer working in the clinic they were attending. 453 (Cancer 10, Diabetes 146, Hypertension 185, and Paediatrics 112) patients were followed up successfully. The summary of patients’ follow-up distribution per clinic is shown in (**Table 2**).

**Table 2:** Summary of patients’ distribution

Clinic	Missed appointments		Qualified follow-up	Followed-up	Not followed up	Referred to nearby facility	Reviewed at referral facility
Cancer		76	12	10	2	None	None
Diabetes		315	212	146	66	48	27
Hypertension		448	293	185	108	89	55
Paediatrics		360	229	112	117	None	None
Total		1199	746	453	293	137	82

**Figure 5:**
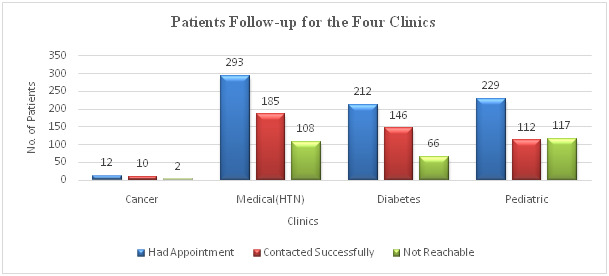
Patients’ follow-up for the four clinics.

293 patients (39.2%) were not followed up successfully because they were either not reachable because the phone number was wrong, or they did not answer the calls. Patients from Cancer (10) and Paediatrics (112) clinics were not referred to other facilities because Machakos Level 5 is the only government facility in Machakos County with a dedicated and a well-equipped cancer centre. For the paediatrics clinics, patients sought health care elsewhere since children are more sensitive and cannot wait for long. Also, during the focus group discussions (FGDs), it emerged that all level 4 facilities had paediatricians and were easily accessible by patients. Out of the remaining 331 patients, 191 (58%) patients (98 from diabetes, and 93 from hypertension) chose Machakos Level 5 Hospital as their preferred facility. Three (1%) deaths were reported from the hypertension clinic and 137 (41%) patients (48 from diabetes, and 89 from hypertension) were referred to other facilities. 82 (60%) patients out of the 137 were reviewed jointly at the referred facilities and the distribution of the referred patients for both clinics is shown in (**Table 3**). The patients' follow-up for the four selected clinics is illustrated in (**Figure 5**).

**Table 3:** Diabetes clinic and hypertension clinic follow-up

Facility	No. of diabetes patients	No. of hypertensive patients
Kangundo Level 4	1	5
Kathiani Level 4	8	20
Matuu Level 4	1	4
Mwala Level 4	9	22
Masinga Level 4	0	2
Athi River Level 3	3	9
Muumandu Level 4	7	6
Mutituni Level 3	19	21
Machakos Level 5	98	93
Nguluni	0	0

**Figure 6:**
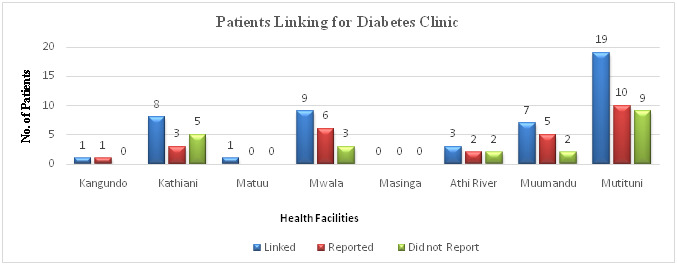
Patients linking for diabetes clinic.

The next step after follow-up involved referral of patients to their preferred or nearest facilities to their homes. On the appointment day, the clinician/physician at the referred facility and the specialist at Machakos Level 5 Hospital connected on phone to review the patient jointly. 82 (60%) patients (27 from diabetes and 55 from hypertension clinic) were reviewed successfully. The remaining 55 (40.1%) patients (21 from diabetes and 34 from hypertension) were not reviewed because either they failed to report to the referral facility, or their files were missing at the registry at the time of linking. Some patients who failed to report at the referred facilities changed their minds and chose Machakos Level 5 as their preferred facility, while others had visited either Machakos Level 5 or the referral facility before the reporting date. The distribution of diabetes patients in the referral facilities is as shown in (**Figure 6**).

**Figure 7:**
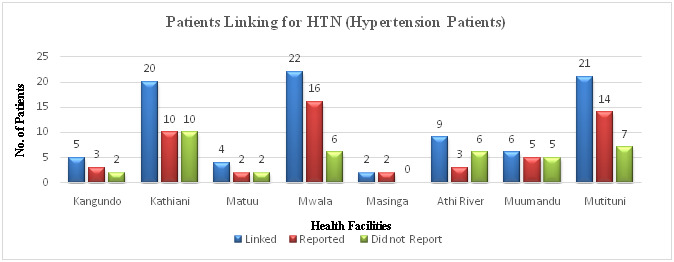
Patients linking for medical (hypertension) clinic.

Out of the 27 patients from the diabetes clinic who were reviewed, 6 (22.2%) reported elevated blood glucose and high blood pressure due to missing clinic appointments. In cases where a patient was taking multiple drugs at the same time, some had missed taking one of the main drugs thus triggering elevation of either blood glucose or blood pressure. Some of the patients reported general body pains, numbness, abdominal discomfort, reflex, vitiligo, and peripheral neuropathy. Two (7.4%) patients were referred for further tests since their outcomes were not good. The remaining 19 (70.4%) patients reviewed were stable reporting well-controlled blood glucose and blood pressure. They had drugs refilled and were scheduled for the next appointment. The distribution of hypertension patients in the referral facilities is as shown in (**Figure 7**).

Out of the 55 patients from the hypertension clinic who were reviewed, 20 (37.7%) reported very highly elevated blood pressure due to missing clinic appointments, not taking all the medications due to drug depletion, delays in getting drug refills, and lack of knowledge on where or when to get refills. Some of the complaints reported by patients included severe headaches, joint pains, numbness, and eye problems. Seven (13.2%) patients were referred for further tests after the review such as renal function tests, Echo tests, electrocardiogram (ECG) tests, magnetic resonance imaging (MRI), lumbar x-ray, laboratory tests, and Cardio checks for irregular pulses and abnormal heart sounds. One (1.9%) patient was referred to Machakos Level 5 because he had a history of Transient Ischemic Attack (TIA) in 2019 and was reporting chest pain and a mild angina pectoris was suspected. The remaining 27 (47.2%) of the reviewed patients, were in stable conditions reporting controlled levels of blood pressure, these patients had their drugs refilled, and given a next appointment date.

## DISCUSSION

From the findings, it is clear that the measures to curb the spread of COVID-19 put in place by the government affected the provision of health care services in Machakos County. Our study aimed to provide tele medical assistance to the patients who missed clinics at the health facilities closest to them. Although statistical analysis could be applied, the main purpose here was to report the success of using telemedicine to extend health services to those patients who could otherwise not have missed such services. From the 1199 patient files reviewed, 977 (81.5%) patients had missed their scheduled appointments, and this was due to fear of contracting COVID-19. The measures put in place by the Kenyan government to curb the spread of COVID-19 such as imposed curfews increased costs of transport, and travel restrictions limited the ability to travel to referral health facilities located far away from patients’ residences. Also, suspension of selected services including specialty clinics at Machakos Level 5 Referral facility to avoid overcrowding at the facility. The cancer clinic recorded the least number of patients because it began operation in September 2019 and was only 7 months old by the onset of the COVID-19 pandemic in Kenya. No referrals were done for the cancer clinic because the cancer clinic was not suspended unlike the other clinics so most of the patients continued to seek health care. Moreover, Machakos Level 5 is the only government facility in Machakos County with a dedicated and well-equipped cancer centre. Few patients 12 (15.7%) had missed the appointment and this was because the cancer clinic was still operational at the time of the study, unlike the other three clinics, which had been suspended.

Out of the 331 patients, followed-up in diabetes and hypertension clinics, 191 (58%) patients chose Machakos Level 5 Hospital as their preferred facility because of ease of access, availability of drugs, well-equipped laboratories for testing, and general confidence in the services at the facility with a high number of specialists. From the diabetes clinic, 48 patients were referred to other facilities, 19 (39.5%) patients chose Mutituni. This could be due to its proximity to Machakos Level 5 Hospital hence most patients residing within its locality would easily access services at Level 5. Only one patient chose Kangundo Level 4 as their nearest facility possibly because this hospital has more specialty clinics compared to other levels 4 and 3 facilities; hence, a majority of patients within its catchment area might not need to seek treatment at Machakos Level 5 hospital. Unlike patients from the diabetes clinic, Kathiani, Mutituni, and Mwala were preferred by an almost equal number of hypertension patients. All these facilities are level 4, managed by medical officers with some being specialists, and can carry out most of the requested laboratory tests apart from ECG. However, they have fewer specialists, and this may have contributed to patients’ seeking services at Machakos Level 5. The proximity of these facilities to Machakos Level 5 Hospital also contributed to the high number of patients naming them as the nearest facility. Kangundo level 4 with a higher number of specialists and offering more specialty clinics compared to the other facilities recorded fewer patients from within its catchment area that sought treatment at Machakos Level 5.

The patients' files that could not be traced at the time of linking were attributed to some patients having multiple comorbidities that required file transfers to other clinics or the wards in case of admissions. Besides, since the hospital lacks an automated file tracking system it was not easy to trace the files when transferred. Most patients 117 (52%) in the Paediatrics clinic were not followed up successfully because of the poor recording of patient information on the patient’s file, poor network coverage, some patients having only one contact record, and next of kin contact missing. This resulted in low response rates and hence the fewer numbers of patients who were followed up successfully compared to the other three clinics where the majority had both patients contact and next of kin. However, linking was not done for this clinic because patients sought health care elsewhere since children are more sensitive and could not wait for long. During the FGDs, it emerged that all level 4 facilities had paediatricians and were easily accessible by patients; as a result, it was not necessary to link the patients to lower facilities.

## CONCLUSIONS AND RECOMMENDATIONS

Due to the automation of the data collection tools using the ODK collect, we were able to collect data faster and this tool enabled offline data collection especially in areas with poor network connectivity. Using the COVID-19 Machakos App expedited the process of patients' follow-up and referral to the nearest facilities, capturing of information in real-time and updating the data to the database. At the referral facility, the mobile App facilitated the tracking of patients and enabled the doctors to give feedback on whether the patients reported to the referred facilities or not. Besides, after reviewing the patients the doctors were able to capture the doctors’ notes on patients' status while ensuring confidentiality and privacy. The Interactive dashboard facilitated the timely generation of analytics reports and summaries through which we were able to observe the patient trends, clinic attendance, and overall provision of health care services during this pandemic period.

This intervention was very timely for we recorded several cases of patients who had elevated blood pressure and sugar levels due to lack of medication, or the dosage of drugs they were taking was not enough. Through this intervention, the patients were reviewed and given care but if they had stayed longer without medication, they would have reported worse outcomes. This study recommends the adoption of technology in the provision of health care as this is evident from the feedback received from the health workers and patients in the referred facilities. The patients were very pleased with this intervention since it saved them time, high transport costs, and the need to travel long distances to seek health care. The joint review of patients with specialists at Machakos Level 5 promoted knowledge transfer since health workers in the smaller facilities acquired vital knowledge that improved the management of patients. The study was conducted in Machakos Level 5 hospital since it the main referral hospital in Machakos County. This facility has the greatest number of consultants and provides most of the specialty clinics thus any interruption to the provision of health care in this facility can result in adverse effects. The authors are appreciative of the fact that the study provides good evidence on how telemedicine can be useful in mitigating worse patient outcomes. However future research that compares different telemedicine approaches could provide additional insights and tools for improved public health management of patients with chronic diseases and could be a subject of future research.

## References

[1] World Health Organization. Coronavirus: Events as they happen. World Health Organisation, 2020. Available: https://www.who.int/emergencies/diseases/novel-coronavirus-2019/events-as-they-happen. Accessed: 10 June 2021.

[2] Khan M Khan H Khan S Nawaz M Epidemiological and clinical characteristics of coronavirus disease (COVID-19) cases at a screening clinic during the early outbreak period: a single-centre study. J Med Microbiol. 2020;69:1114-23. 10.1099/jmm.0.001231 32783802 PMC7642977

[3] Special Expert Group for Control of the Epidemic of COVID-19 of the Chinese Preventive Medicine Association Consideration on the strategies during epidemic stage changing from emergency response to continuous prevention and control. Zhonghua Liu Xing Bing Xue Za Zhi. 2020;41:297-300. 32088947 10.3760/cma.j.issn.0254-6450.2020.03.003

[4] Worldometer. COVID Live Update: Cases and Deaths from the Coronavirus. 2020. Available: https://www.worldometers.info/coronavirus/. Accessed: 10 August 2020.

[5] Ministry of Health. First case of coronavirus disease confirmed in Kenya. Press Release. 2020. Available: https://www.health.go.ke/wp-content/uploads/2020/06/UPDATE-ON-CORONAVIRUS-6TH-JUNE-2020.pdf. Accessed: 10 June 2021.

[6] Government of Kenya. Address to the nation by h.e. uhuru kenyatta, c.g.h, president of the republic of kenya and commander-in-chief of the defence forces on covid-19, commonly known as coronavirus at harambee house, nairobi on 15th march 2020 | the presidency. 2020. Available: https://www.president.go.ke/2020/03/15/address-to-the-nation-by-h-e-uhuru-kenyatta-c-g-h-president-of-the-republic-of-kenya-and-commander-in-chief-of-the-defence-forces-on-COVID-19-commonly-known-as-coronavirus/. Accessed: 10 June 2021.

[7] Mwangi N. Kenya recorded highest daily spike in COVID-19 cases as tally passes 15,000. CGTN Africa. 2020. Available: http://www.xinhuanet.com/english/africa/2020-07/24/c_139235746.htm. Accessed: 10 June 2021.

[8] Zhu A. Briefing: Five ideas on how to ease the impact of COVID -19 lockdowns in Kenya one another. The New Humanitarian, 2020. Available: https://www.thenewhumanitarian.org/news/2020/04/06/kenya-coronavirus-lockdowns. Accessed: 10 June 2021.

[9] World Bank. Socioeconomic Impacts of COVID-19 in Kenya On Households. Washington DC: World Bank; 2021.

[10] Aljazeera. COVID-19: Kenya bans travel in and out of Nairobi, other areas. Aljazeera. 2020. Available: https://www.aljazeera.com/news/2020/4/6/COVID-19-kenya-bans-travel-in-and-out-of-nairobi-other-areas. Accessed: 10 June 2021.

[11] UNAIDS. The cost of inaction: COVID-19-related service disruptions could cause hundreds of thousands of extra deaths from HIV. UNAIDS Press Release. 2020. Available: https://www.who.int/news/item/11-05-2020-the-cost-of-inaction-COVID-19-related-service-disruptions-could-cause-hundreds-of-thousands-of-extra-deaths-from-hiv%0Ahttps://www.unaids.org/en/resources/presscentre/pressreleaseandstatementarchive/2020/may/202005. Accessed: 10 June 2021.

[12] Bashshur RL Reardon TG Shannon GW Telemedicine: A new health care delivery system. Annu Rev Public Health. 2000;21:613-37. 10.1146/annurev.publhealth.21.1.613 10884967

[13] Bashshur R Doarn CR Frenk JM Kvedar JC Woolliscroft JO Telemedicine and the COVID-19 Pandemic, Lessons for the Future. Telemed J E Health. 2020;26:571-73. 10.1089/tmj.2020.29040.rb 32275485

[14] Siwicki B. Telemedicine during COVID-19: Benefits, limitations, burdens, adaptation | Healthcare IT News. health care it news, 2020. Available: https://www.healthcareitnews.com/news/telemedicine-during-COVID-19-benefits-limitations-burdens-adaptation. Accessed: 10 June 2021.

[15] Centers for Disease Control and Prevention (CDC). Using Telehealth to Expand Access to Essential Health Services during the COVID-19 Pandemic. Centers for Disease Control and Prevention. Available: https://www.cdc.gov/coronavirus/2019-ncov/hcp/telehealth.html#anchor_1591720077356%0Ahttps://www.cdc.gov/coronavirus/2019-ncov/hcp/telehealth.html%0Ahttps://www.cdc.gov/coronavirus/2019-ncov/hcp/telehealth.html#edn10%0Ahttps://www.cdc.gov/coronavirus/2019. Accessed: 10 June 2021.

[16] East African Community. East African Community Health Sector Investment Priorities Framework 2018 – 2028. Available: https://health.eac.int/file-download/download/public/278. Accessed: 10 June 2021.

